# Tool Wear Effect on Machinability and Surface Integrity in MQL and Cryogenic Hard Turning of AISI 4340

**DOI:** 10.3390/ma18235423

**Published:** 2025-12-02

**Authors:** Nabil Jouini, Saima Yaqoob, Jaharah A. Ghani, Sadok Mehrez

**Affiliations:** 1 Mechanical Engineering Department, College of Engineering, Prince Sattam Bin Abdulaziz University, Alkharj 11942, Saudi Arabia; s.mehrez@psau.edu.sa; 2 Department of Mechanical and Manufacturing Engineering, Faculty of Engineering and Built Environment, Universiti Kebangsaan Malaysia, Bangi 43600, Selangor, Malaysia; engg_saima@hotmail.com (S.Y.); jaharahaghani@ukm.edu.my (J.A.G.); 3 Department of Industrial and Manufacturing Engineering, NED University of Engineering and Technology, Karachi 75270, Pakistan

**Keywords:** cutting force, chip temperature, surface roughness, microstructure alteration, cryogenic (LN_2_), minimum quantity lubrication (MQL)

## Abstract

Hard turning has emerged as a cost-effective and flexible alternative to conventional grinding for machining hardened steels such as AISI 4340. However, its performance is significantly influenced by the choice of cooling and lubrication strategies, as well as the condition of the cutting tool. Inadequate thermal management and tool wear can lead to elevated cutting forces, high interface temperatures, degraded surface quality, and an altered microstructure. This study investigates the machinability performance of AISI 4340 alloy steel (50 HRC) using CVD-coated carbide tools under two distinct cooling/lubrication environments: minimum quantity lubrication (MQL) and cryogenic cooling (LN_2_). Experiments were conducted at the beginning and end of tool life with both environments to capture the influence of tool wear on key performance indicators, including cutting force, chip temperature, surface roughness, and microstructural integrity. Results indicate that LN_2_ cooling outperformed MQL in mitigating thermal loads and maintaining surface quality, particularly under worn tool conditions. LN_2_ reduced cutting forces by up to 37.10%, chip temperature by 56.68%, and surface roughness by 36.95% compared to MQL. Microstructural analysis revealed significantly thinner deformation and white layers under LN_2_, suggesting improved subsurface integrity. These findings highlight the potential of LN_2_ cooling for enhancing the machinability of hard turning operation and improving overall performance in industrial applications.

## 1. Introduction

Machining is a value-added process that transforms raw materials into finished products. With the advent of advancements in manufacturing technologies, several modern and cost-effective strategies have increasingly replaced traditional practices [1]. For instance, the machining of hardened materials was conventionally performed through a grinding process, which typically required a series of preliminary steps including forming, annealing, rough machining, heat treatment, and final grinding. This multi-stage approach led to prolonged cycle times and increased manufacturing costs [2]. In contrast, hard turning has emerged as a viable alternative due to its capability to machine hardened materials in fewer steps, thereby reducing processing time and operational complexity. However, a major limitation of hard turning is the accelerated rate of tool wear, which can potentially increase the overall manufacturing cost [3]. To address this, manufacturers are increasingly adopting more efficient and environmentally friendly machining practices [4]. Among these, minimum quantity lubrication (MQL) and cryogenic cooling have shown considerable promise in mitigating tool wear while maintaining or even surpassing the surface quality achieved through conventional grinding processes [5]. Studies have reported that hard turning, particularly when assisted with advanced tools and cooling/lubrication techniques, offers a sustainable alternative by minimizing the use of hazardous cutting fluids, thereby reducing environmental impact [1]. Moreover, these methods have demonstrated improved performance in terms of tool life, cutting force reduction, and enhanced surface finish [6].

The hard turning of AISI 4340 alloy steel using cermet tools has been shown to achieve surface roughness (Ra) ranging from 0.212 to 1.452 µm, which are values considerably below the acceptable grinding threshold of 1.6 µm. This justifies the utilization of advanced cutting tool materials even at increasing cutting speeds (70–140 m/min) and feed rates (0.05–0.2 mm/rev) [7]. Similarly, studies using CBN tools have also reported exceptional surface quality, with a minimum Ra of 1.8 µm in hard turning alloy steel [8]. Sato et al. [9] compared hard turning under MQL conditions with conventional grinding and found that the former offered superior performance, including a 29% reduction in surface roughness, 9% improvement in dimensional accuracy, 127% increase in tool life, 13% reduction in energy consumption, and 42% decrease in acoustic emission. Moreover, cryogenic cooling using a vortex tube has also demonstrated superior results during the hard turning of AISI 4340, with a notable 36.30% reduction in cutting force compared to MQL. This improvement was attributed to the tabular chip morphology generated under cryogenic conditions, which reduced friction at the tool–chip interface and facilitated smoother chip evacuation.

Khosravi et al. [10] examined the influence of the following cutting parameters: cutting speed, feed rate, depth of cut, and tool radius on temperature and surface roughness during turning of AISI 4340. Their integrated approach, combining experimental trials, finite element modeling, and response surface methodology (RSM), identified optimal parameters to minimize simultaneously surface roughness and temperature. In another study, Kumar and Raj [11] explored the effects of a textured rake face on the cutting performance. They observed that moderately textured tools significantly reduced cutting forces and temperatures while improving chip flow, although excessively deep grooves had the opposite effect. Likewise, Singh et al. [12] further evaluated the use of canola oil and graphene-enhanced canola oil with textured tools under MQL conditions, employing single jet and twin jet nozzle configurations. The twin-jet system, when used with optimally textured tools, resulted in lower cutting forces, enhanced dimensional accuracy, and reduced energy consumption.

Cutting temperature and cutting force are critical parameters that directly influence the functional performance of machined components. Higher levels of either can lead to detrimental surface and subsurface defects, including high surface roughness, white layer formation, smearing, and thermal cracking, all of which can severely reduce the fatigue life of the material [13]. In a recent investigation, Abedrabbo et al. [14] demonstrated that LN_2_ machining of heat-treated AISI 4340 significantly minimized surface defects compared to conventional wet machining, including a reduction in the thermally affected layer, the white layer, the punctual defect, and in surface irregularities. White layers are generally undesirable due to their brittle nature and susceptibility to crack propagation [15]. However, Abedrabbo et al. [14] stated that if the thickness of machining-induced defects, including the white layer, is less than 4 µm, the material properties of the machined component are not compromised. According to Ref. [16], elevated stress/strain rates and temperatures during hard turning contribute to both mechanically and thermally induced white layer formation. Mechanically induced white layers form below the austenitizing temperature due to severe plastic deformation, while thermally induced layers occur above this temperature due to alternating heating and cooling cycles. Several studies have focused on minimizing both white layer and plastic deformation thickness. For instance, Sivaiah and Chakradhar [17] reported that cryogenic machining of 17-4 PH stainless steel resulted in the lowest white layer thickness (0.737 µm), compared to dry (2.02 µm), wet (1.827 µm), and MQL (1.376 µm) conditions. This was attributed to reduced plastic deformation and enhanced cooling effects under LN_2_. Similarly, gas-based cooling during the hard turning of D4 steel was found to promote effective vaporization, reduce heat generation, and enhance heat dissipation, thus aiding in the suppression of white layer formation [18].

Despite the considerable literature on improving hard turning performance using various cutting tools and machining environments, most existing studies primarily focus on tool wear and surface roughness. However, detailed investigations into cutting forces and cutting temperatures, key factors influencing surface roughness and microstructural alterations on and beneath the machined surface remain limited, particularly in the context of coated carbide tools used for turning heat-treated AISI 4340 alloy steel under MQL and LN_2_ environments. This study addresses this gap by examining the interplay between tool wear, cutting force, and cutting temperature, and their subsequent effects on surface roughness and subsurface microstructural transformations. The findings provide insights into the deformation mechanisms induced by thermo-mechanical loads under different lubrication conditions. The outcomes are expected to contribute toward assessing the industrial feasibility of these cooling strategies and encouraging further research on enhancing the cost-effectiveness and performance of coated carbide tools in hard turning applications.

## 2. Materials and Methods

The machining experiments were conducted on, manufactured by 600 Group, UK. Cylindrical AISI 4340 alloy steel bars were selected due to their widespread use in manufacturing critical components. The specimens were prepared with a length of 120 mm and a diameter of 60 mm, maintaining an L/D ratio < 10 in accordance with the previous literature [19] to minimize vibration and ensure stable cutting conditions. Prior to machining, the samples underwent heat treatment comprising austenitizing, quenching, and tempering to achieve a hardness of approximately 50 HRC.

Commercially available ISO-P20 CVD-coated carbide inserts (Al_2_O_3_/TiCN) from Sumitomo Electric (Japan) were used to conduct the experiments. The selected insert had a CNMG 120404 geometry and was mounted on a DCLNR 2020K 12 tool holder, by Sandvick Coromant, UK resulting in a clearance angle of 6°, a rake angle of −6°, and a tool nose radius of 0.4 mm. The tool featured a multilayer coating with a total thickness of 18 µm, consisting of an inner TiCN layer and an outer Al_2_O_3_ layer. Experiments were carried out once under two distinct environments: minimum quantity lubrication (MQL) and cryogenic cooling using liquid nitrogen (LN_2_). For MQL, a commercially available lubricant (Coolube 2210XP) by UNIST Inc. USA was applied through a dual-nozzle system, one directed at the rake face and the other at the flank face of the tool. This dual-nozzle configuration was adopted based on previous studies [20,21,22], which demonstrated superior performance compared to single-nozzle systems due to the improved boundary lubrication at both the tool–chip and tool–workpiece interfaces. The flow rate for MQL was maintained at 60 mL/h, with both nozzles oriented at 45° angles and positioned 30 mm from the tool, as recommended in prior studies on alloy steels [23,24].

**Figure 1:**
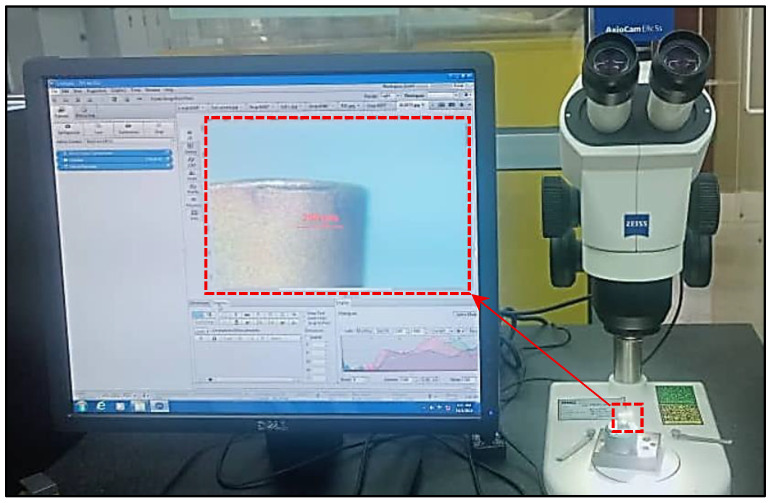
Measurement of tool flank wear (Vb) using optical microscope.

In cryogenic machining, the LN_2_ jet was delivered from a copper nozzle onto the tool–chip interface, following the approach of Ref. [25], to reduce the secondary deformation temperature. The nozzle was directed exclusively at the rake face to lower chip temperature and facilitate its removal, while avoiding the flank face, as the delivery of LN_2_ from flank face has been reported to increase material hardness and promote subsurface hardening [26]. Machining experiments under both cooling/lubrication conditions were conducted using a fresh tool and continued until the average flank wear (Vb) reached 300 µm, in accordance with ISO 3685. Tool wear was measured periodically every 2 min using a Zeiss Stemi 2000 optical microscope produced by Germany equipped with integrated software ZEN 2012 SP2 for image capture and dimensional measurement. Periodic measurements continued until the tool life criterion was reached. The microscopic image captured using the Zeiss Stemi 2000 microscope can be seen in Figure 1. The cutting parameters, including cutting speed (V) = 300 m/min, feed rate (f) = 0.05 mm/rev, and depth of cut (doc) = 0.1 mm, were selected based on optimized conditions reported by Jouini et al. [27] for the hard turning of AISI 4340 using same coated carbide tools. The details of machining parameters and response variables are presented in Table 1. These machining parameters were selected based on the result at optimum conditions from this previous study [27].

Response variables including cutting force, chip temperature, surface roughness, and microstructural alterations were evaluated. Cutting forces were measured using the NeoMomac system, developed in-house at Universiti Kebangsaan Malaysia [28]. The average values of all three force components, cutting force ${F_{x}}^{2},$ radial ${F_{r}}^{2}$, and axial ${F_{a}}^{2}$, were recorded for the respective machining pass and the resultant force was calculated using vector summation for analysis by Equation (1).

(1) $$F_{c}=\sqrt{{F_{x}}^{2}+{F_{r}}^{2}+{F_{a}}^{2}}$$

Chip temperature was monitored using the G100EX NEC Thermo Gear thermal infrared camera, manufactured by Nippon Avionics Co., Ltd. Japan with a resolution of 2 megapixels, capable of capturing the high-temperature regions of moving targets. The camera is capable of capturing moving objects and can record maximum of 6000 frames at a 10 Hz frame rate during cutting. A distance of approximately 1 m was maintained between the camera and the workpiece, and an emissivity value of 0.1 was used based on the workpiece material (unoxidized steel), following the equipment manual. Thermal images were exported to InfRec Analyzer NS9500 Lite software Ver.7.1D, and the maximum temperature in the respective frame was identified and used for analysis. Surface roughness measurements were performed using a Mitutoyo Surftest SJ-210 profilometer by Mitutoyo Japan. Readings were taken at three different angular positions (separated by 120°) for each sample under both fresh and worn tool conditions across both environments. The average Ra value was reported for comparison.

**Figure 2:**
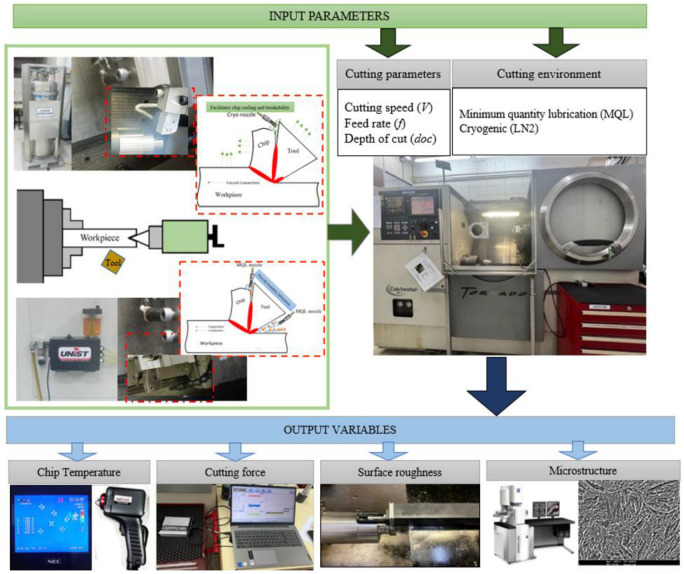
Schematics of experimental setup for hard turning.

Following the machining experiments, the cross-sections of the samples were sectioned using wire-cut EDM to minimize thermal damage. The specimens were hot mounted and then subjected to sequential grinding using abrasive papers with grit sizes of 200, 400, 600, 800, and 1200 (coarse and fine). Polishing was performed using diamond lubricant and ultra-fine diamond suspension until a mirror-like surface was achieved. The samples were then etched using 2% nital solution for 15–20 s to reveal the microstructure. Microstructural and subsurface analyses were conducted using a ZEISS field emission scanning electron microscope (FESEM) by ZESIS Germany, Marlin model, capable of magnifications ranging from 12× to 2,000,000×. High-resolution images were captured to examine surface and subsurface deformation, white layer formation, and grain refinement. Figure 2 shows the experimental setup of the turning tests.

## 3. Results and Discussion

The machining experiments were carried out using fresh tools under both MQL and LN_2_ environments. Measurements taken for the fresh tool represent the initial condition, with flank wear (Vb) equal to 0 µm. During continuous machining, tool wear progressed, and Vb was monitored using an optical microscope at 2 min intervals until the tool life criterion was reached. In this study, the worn tool refers to the measurements taken at the final pass. In the final pass, the flank wear reached 346.62 µm for MQL and 310.78 µm for LN_2_. It was observed that after reaching approximately 250 µm, the wear under MQL conditions progressed more rapidly, leading to severe wear and ultimately reaching the tool life criterion with a higher flank wear value compared to LN_2_. Table 2 shows the results obtained for this study.

### 3.1. Resultant Cutting Force (F_c_)

**Figure 3:**
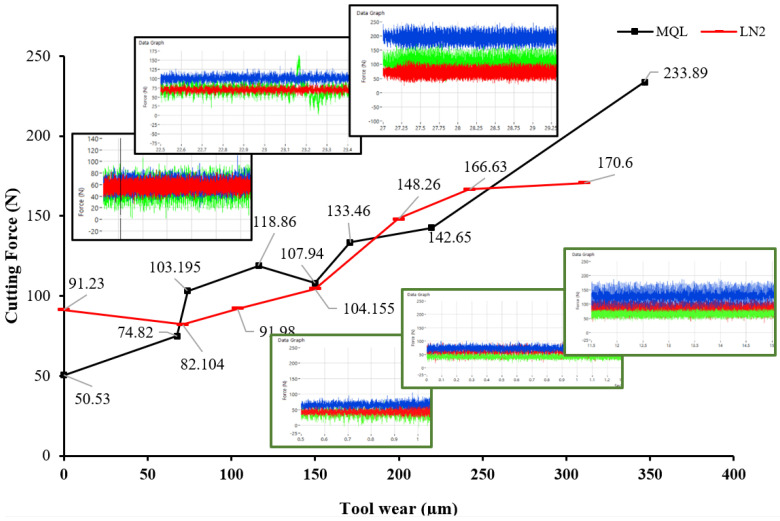
Cutting force variation with progressing tool wear under MQL and LN_2_ environments.

Measuring cutting force (F_c_) is critical, as it offers valuable insights into tools’ performance, energy consumption, and mechanical stresses on the cutting edge, all of which are essential for optimizing the machining process and extending tool life [29]. Previous studies have reported that the application of cooling and lubrications techniques can favorably reduce the coefficient of friction, thereby decreasing the required cutting force [30]. Accordingly, this section comprehensively discusses the influence of various machining conditions on the reduction in resultant cutting force. The results of the cutting force with different machining and tool wear states are mentioned in Table 2. Figure 3 shows the trend of resultant cutting force as a function of tool wear under MQL and LN_2_ environments. It was observed that the cutting force measured under MQL conditions was significantly lower (50.53 N) compared to LN_2_ (91.23 N) cooling. This corresponds to an approximate increase of 80.55% in cutting force when using LN_2_ at the initial tool wear stage. It can be stated that the increase in cutting force with LN_2_ is attributed to the strain hardening effect induced by low temperature cryogenic coolants, as reported in previous studies [31,32]. On the other hand, application of micro-lubricating oil during the initial passes of cutting provided boundary lubrication film which reduced the coefficient of friction and heat generation during the early stage of machining, as supported by the findings of Mallick et al. [33] on the hard turning of AISI D2 steel under a dual-nozzle MQL system.

A general upward trend in cutting force is observed for both environmental strategies as the tool wear progresses from a fresh to severely worn state. This observation aligns with the established literature, which reports that increasing tool flank wear leads to higher friction, a loss of effective rake angle, and a greater cutting resistance at the tool–workpiece interface [34]. During the intermediate stages of tool wear both environments exhibit fluctuations in cutting force. However, the rise in force under LN_2_ is comparatively steadier and exhibits lower peak variations, likely due to its superior cooling capacity, which suppresses the thermal softening of the tool, preserves cutting edge integrity, and reduces adhesion-related forces [35]. In contrast, MQL shows more pronounced signal noise and force peaks, especially at a higher wear state, suggesting more erratic cutting behavior resulting from edge chipping, flank adhesion, and unstable contact conditions. These findings are consistent with the results of Iqbal et al. [36] who conducted a comparative analysis of single- and multi-pass machining under MQL and LN_2_ environments. Their study revealed that double-pass cryogenic machining resulted in a 6.66% reduction in cutting energy consumption compared to MQL. Toward the end of tool life, the limited cooling effectiveness of MQL further deteriorated its performance, leading to a 37.10% higher cutting force compared to LN_2_ cooling at the final wear stage.

### 3.2. Cutting Temperature

**Figure 4:**
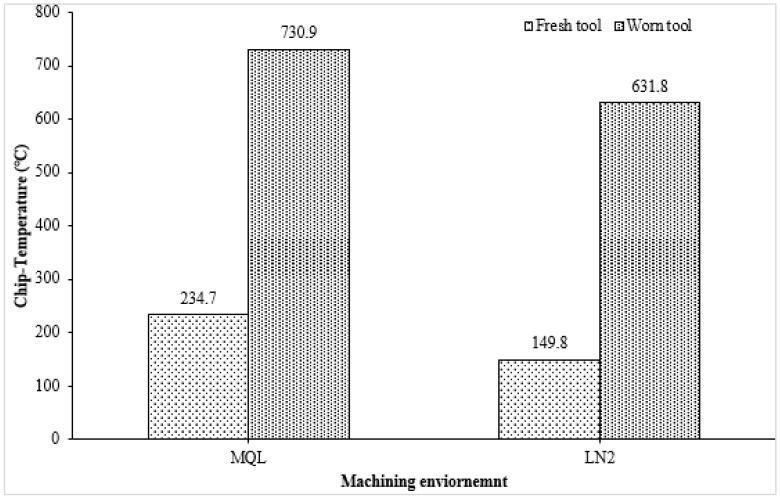
Chip temperature recorded for MQL and LN_2_ environments with fresh and worn tools.

**Figure 5:**
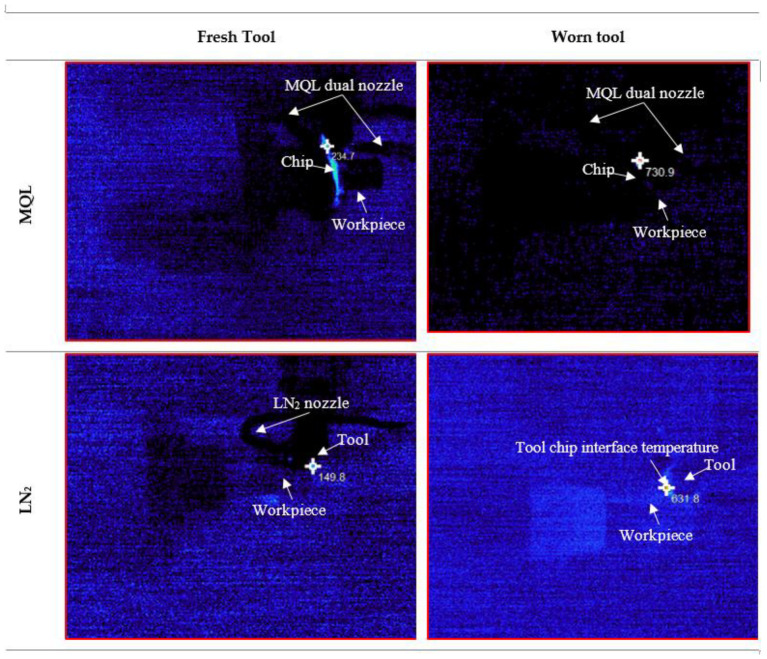
Images recoded from thermo Gear thermal infrared camera for MQL and LN_2_ environments.

Cutting temperature measurement is critically important in machining operations due to its direct influence on tool performance, workpiece integrity, and overall process efficiency [10]. The accurate measurement and control of cutting temperature are therefore essential for optimizing process parameters, selecting appropriate cooling/lubrication techniques, and ensuring consistent quality and sustainability in high-precision manufacturing [35]. Figure 4 and Figure 5 present the comparative assessment of chip temperature under MQL and LN_2_ machining environments for both fresh and worn tools. The result clearly indicates that cryogenic cooling significantly reduced the cutting temperature compared to MQL. For instance, the chip temperature with the fresh tool under LN_2_ was 149.8 °C, while under MQL it was 234.7 °C, which is approximately 56.68% higher. This substantial reduction is attributed to LN_2_’s extremely low boiling point (−196 °C), which enables rapid heat absorption, thereby limiting thermal accumulation in the heat-affected zone [37]. Furthermore, LN_2_ effectiveness enhanced when delivered from the rake face of the cutting tool. In this configuration, the coolant directly interacts with the tool–chip interface, rapidly evaporating and absorbing the interface heat [38]. Additionally, LN_2_ projected from the rake side facilitates chip curling and smoother chip evacuation from the deformation zone, which reduces the coefficient of friction and the overall cutting temperature [4].

Furthermore, the observed increase in chip temperature with progressive tool wear in both environments is consistent with established machining principles. As the tool becomes worn, the contact area enlarges and the cutting edge blunts, leading to greater plastic deformation and friction, thereby elevating the thermal load [39]. Nevertheless, even under worn tool conditions, LN_2_ exhibited superior performance by maintaining a 13.56% lower chip temperature compared to MQL. The reduced thermal control of MQL in this context can be attributed to the limited cooling capacity of micro-lubrication. At higher tool wear levels, the burning or evaporation of the lubricant, especially if the cutting temperature exceeds the flash point of the oil, can compromise its effectiveness. As reported by Ref. [40], if the flash point of the lubricant is lower than the machining temperature, MQL performance deteriorates significantly. Consequently, the poor performance of commercially utilized MQL in terms of tool life may be linked to its relatively low flash point (~200 °C), which undermines its lubrication effectiveness under elevated thermal conditions, ultimately accelerating tool wear.

### 3.3. Surface Integrity

#### 3.3.1. Surface Roughness

**Figure 6:**
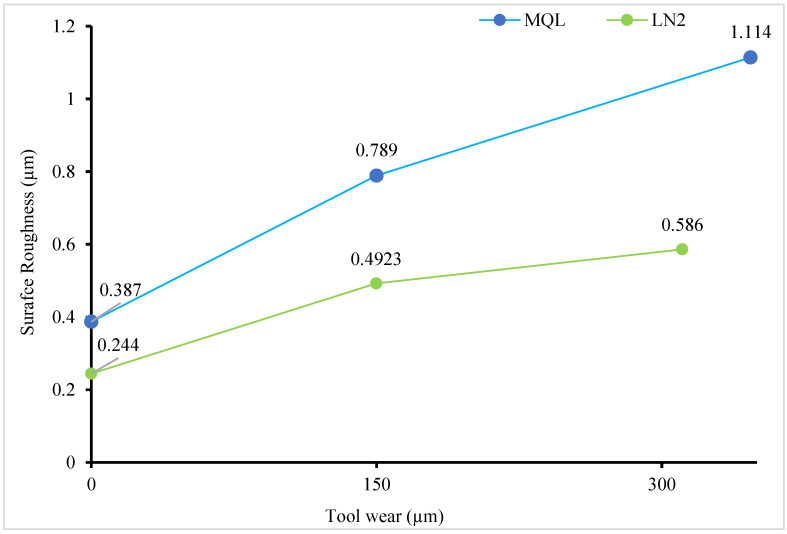
Surface roughness variation with tool wear for MQL and LN_2_ environments.

It is imperative to monitor surface roughness during machining, as tribological interactions between tool–workpiece interfaces can markedly alter the cutting edge geometry, potentially inducing tool vibration, increasing cutting forces, and thereby compromising the surface integrity of the machined component [41]. Table 2 shows the value of average surface roughness with MQL and LN_2_ conditions. The lowest surface roughness of 0.244 µm was observed with the fresh tool under LN_2_ cooling, as illustrated in Figure 6. In comparison, MQL exhibited a considerably higher value, representing an approximate increase of 58.60% over LN_2_. Notably, the outperformance achieved with LN_2_ cooling is comparable to that obtained using the advanced cutting tool reported in previous studies. For example, Da Silva et al. [8] reported Ra values of 0.18 µm and 0.22 µm using PCBN and ceramic tools at v = 300 m/min, f = 0.05 mm/rev, and d = 0.15 mm during the hard turning of AISI 4340 steel (52 HRC). This is comparable to the result obtained with the fresh tool under cryogenic coolant.

As expected, an increasing trend in surface roughness was observed for both conditions as tool wear progressed from fresh to worn states. This trend aligns with the previously published literature stating that the degradation of the tool coating and the subsequent exposure of the carbide substrate, driven by friction, elevated temperatures and chemical interactions [42]. However, the variation in surface roughness was notably lower under the cryogenic coolant. Even at the end of a tool’s life, LN_2_ demonstrated a 36.95% reduction in surface roughens compared to MQL. Sartori et al. [43] similarly reported the superior performance of LN_2_ in a comparative assessment involving dry, LN_2_, MQL, and LN_2_ + MQL environments.

One contributing factor to the enhanced surface quality achieved with LN_2_ is its ability to generate a high thermal gradient, which suppresses plastic side flow of the material and minimizes surface defects [44]. In contrast, the accelerated increase in tool flank wear observed under MQL is attributed to its limited thermal stability at elevated cutting temperatures. As the lubricant begins to evaporate or burn, the formation of a consistent boundary lubrication film is hindered. Consequently, the reduction in lubrication efficiency leads to increased friction at the tool–workpiece interface, thereby resulting in higher surface roughness values [39].

#### 3.3.2. Microstructure Alteration

**Figure 7:**
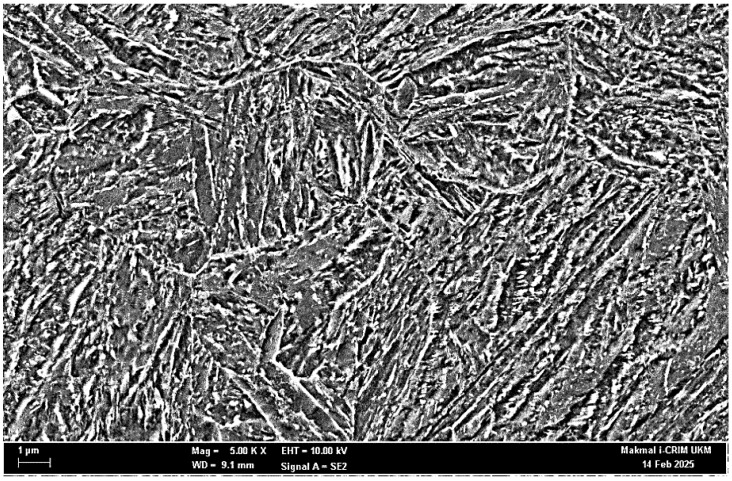
Microstructure of as-received material.

The progressive cutting cycles and corresponding tool wear state exert a significant influence on the microstructural characteristics of the machined surface. This section presents the microstructural alteration induced by varying tool wear conditions in MQL and LN_2_ environments. As depicted in Figure 7, the microstructure of the as-received, heat-treated AISI 4340 alloy steel prior to machining comprises lath martensite, lath blocks, and distinct lath boundaries. However, with continued machining, microstructural transformations were evident, contingent upon the extent of tool wear and the employed cooling/lubrication method. These changes are primarily attributed to the varying thermo-mechanical stresses acting at the cutting interface, which progressively modify the initial microstructure. Muhamad et al. [45] reported that during high-speed machining, severe mechanical deformation can lead to grain elongation and fracture, ultimately resulting in the formation of a nanocrystalline surface layer. Additionally, the degree of thermal exposure and rapid cooling during machining contributes to the development of refined layer (RL) and transition layer (TL), each exhibiting distinct mechanical properties relative to the base material [46].

**Figure 8:**
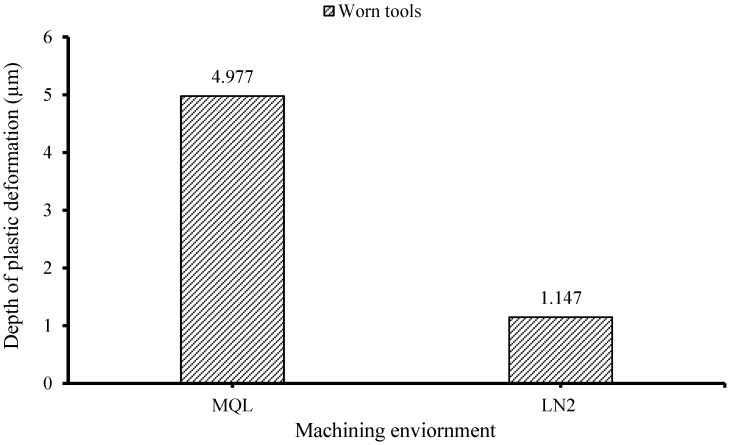
Thickness of plastic deformation with worn tools.

The microstructural alterations on the machined surfaces induced by worn tools under different machining environments are illustrated in Figure 8. Notably, in contrast to the worn tools, no significant microstructural deformation was observed in samples machined with fresh tools. This indicates that the associated thermal and mechanical loads were insufficient to cause notable microstructural modifications within the material. This interpretation is further validated by the substantially lower cutting temperatures and cutting forces recorded under fresh tool conditions. It is also important to clarify that the apparent appearance of white layer on the machined surface is not a true machining-induced feature, but rather an artifact of SEM imaging conditions. This is evident from the magnified view, where the grain structure remains visible in the seemingly white region. Additionally, prior studies have reported that sample preparation using wire-cut EDM may also induce a white-layer-like appearance due to thermal effects during the sectioning process, further emphasizing the need for the careful interpretation of white layer formation.

**Figure 9:**
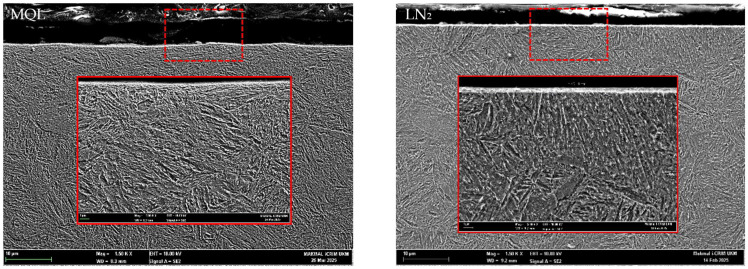
Microstructural morphology of MQL and LN_2_ environments with fresh tools.

**Figure 10:**
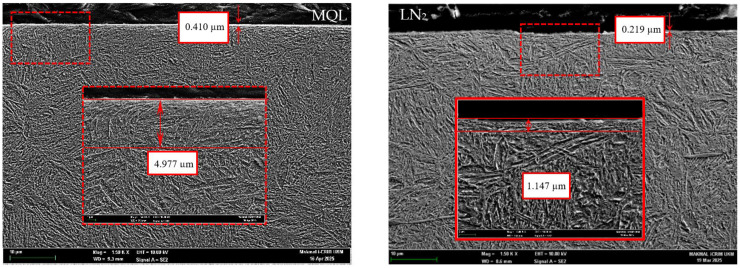
Microstructural morphology of MQL and LN_2_ environments with worn tools.

Figure 9 and Figure 10 present FESEM micrographs of machined samples processed using fresh and worn tools under MQL and LN_2_ conditions. In both cases, visible microstructural deformation is evident in the form of plastic deformation layers or material drag, particularly along the direction of cutting speed. Zhang, Li, and Lv [47] reported that the machined surfaces of heat-treated H13 tool steel subjected to high tool flank wear exhibited extensive subsurface damage, including grain extrusion, crushing, stretching, and the dispersion of carbide particles within the deformed matrix. However, when LN_2_ cooling was employed, the extent of plastic deformation was significantly reduced compared to the MQL condition. This is substantiated by the observed reduction in the deformation layer thickness from 4.977 µm under MQL to 1.147 µm under LN_2_, representing a 76.95% decrease when machining with worn tools. This reduction can be attributed to the reduced thermal–mechanical loads associated with cryogenic machining. As reported earlier, the chip temperatures were notably lower under LN_2_ cooling at 149.8 °C using fresh tools and at 631.8 °C using worn tools, which prevented the thermal softening of the workpiece material, allowing it to retain its hardness during cutting. Consequently, atomic mobility and dislocation activity near the surface LN_2_ aid in preserving the microstructural integrity by limiting the plastic deformation.

In contrast, MQL provided a limited cooling capability, which results in elevated tool–chip interface temperatures, particularly when machining with a worn or blunt cutting edge. This leads to increased friction and cutting forces, along with localized thermal softening of the workpiece surface, thereby promoting microstructural alterations. As a result, a plastically deformed subsurface exhibiting noticeable plastic flow streamlines along the cutting direction, representing severe microstructural distortion. Additionally, white layer formation was observed in these samples with thicknesses of 0.410 µm in MQL and 0.219 µm in LN_2_ conditions. The mechanism behind white layer formation under these conditions can be partly explained by the corresponding chip temperatures. It is hypothesized that under LN_2_ cooling, the cutting temperature at the tool–workpiece interface may not have reached the austenitization temperature of steel (approximately 900 °C) [48]. As a result, there was insufficient thermal energy to enable the atomic diffusion required for the formation of new grains [48]. According to Hosseini and Klement [16], at temperatures below the austenitization point, nano-sized microstructures formed through a mechanism known as dynamic recovery (DRV). This process induced dislocation density and reorganizes the material’s internal structure without forming new grains. Under such conditions, the grains are elongated and gradually break down into substructures due to severe plastic deformation (SPD). This interpretation aligns well with the observed grain morphology under MQL, where the microstructural features are consistent with DRV-induced deformation. Although the recorded chip temperature under MQL (730.9 °C) was relatively high, it may not have been sufficient for complete recrystallization. Thus, it is plausible that the white layer observed under MQL was a combined result of both dynamic recovery and localized dynamic recrystallization, driven by the elevated mechanical and thermal loads during machining with a worn tool.

## 4. Conclusions

The following hard turning of AISI 4340 alloy steel was performed using CVD-coated carbide tools under MQL and LN_2_ conditions to assess their effectiveness with fresh and worn tools. Key machinability aspects, including cutting force, chip temperature, surface roughness, and microstructural alteration, were comprehensively analyzed. The following conclusions can be drawn from the study:

1. The cutting force results with the fresh tool showed a significant reduction in the resultant cutting force under MQL (50.53 N), which was 80.55% lower than that observed under LN_2_ cooling during the initial cutting passes. The higher force in LN_2_ conditions was attributed to strain hardening effects induced by the rapid cooling. However, with worn tool conditions, LN_2_ outperformed MQL by reducing the cutting force to 170.6 N, whereas MQL exhibited erratic cutting behavior with peaks reaching 233.89 N.
2. LN_2_ demonstrated superior thermal management in both fresh and worn tool scenarios. With a fresh tool, the chip temperature under LN_2_ was significantly lower (149.8 °C) compared to MQL (234.7 °C), reflecting a 56.68% reduction. Even with a worn tool, LN_2_ maintained a 13.56% lower chip temperature than MQL. This improvement is attributed to LN_2_’s extremely low boiling point (−196 °C), enabling rapid heat absorption and reduced thermal accumulation in the cutting zone.
3. The lowest surface roughness (0.244 µm) was achieved under LN_2_ cooling with a fresh tool. In comparison, MQL resulted in 58.60% increase in roughness value. Notably, even at the end of tool life, LN_2_ retained its effectiveness, achieving a 36.95% reduction in surface roughness relative to MQL. This is due to MQL’s diminished thermal stability at elevated temperatures, which leads to lubricant evaporation and a subsequent loss of lubrication efficacy.
4. The microstructural examination revealed that worn tools induced more pronounced plastic deformation compared to fresh tools. LN_2_ effectively minimized subsurface deformation, reducing the deformation layer thickness to 1.147 µm, compared to 4.977 µm under MQL, representing a 76.95% reduction. Additionally, thinner white layers were observed under LN_2_ (0.219 µm) compared to MQL (0.410 µm). These results indicate that thermal and mechanical loads during machining significantly influence white layer formation with worn tools. Under LN_2_, the microstructural changes were primarily driven by mechanical effects, while in the MQL condition, both dynamic recovery and localized dynamic recrystallization, induced by combined thermal and mechanical stresses, contributed to the observed alterations.

It is suggested that future studies should evaluate the fatigue performance and residual stress profiles of components machined with both fresh and worn tools under different machining environments. Such analysis would provide deeper insights into how tool wear influences long-term functional behavior, particularly in applications where cyclic loading is critical. Investigating these aspects would help practitioners determine whether machining up to the conventional wear limit is acceptable or whether earlier tool replacement is necessary to ensure part reliability and structural integrity.

## Figures and Tables

**Table 1:** Input machining parameters and cooling/lubrication environments.

Exp	Machining Parameters	Tool State (Vb)	Machining Environment
1	V = 300 m/min, f = 0.05 mm/rev, doc = 0.1 mm	Fresh tool	MQL
2	V = 300 m/min, f = 0.05 mm/rev, doc = 0.1 mm	Fresh tool	Cryogenic (LN_2_)
3	V = 300 m/min, f = 0.05 mm/rev, doc = 0.1 mm	Worn tool	MQL
4	V = 300 m/min, f = 0.05 mm/rev, doc = 0.1 mm	Worn tool	Cryogenic (LN_2_)

**Table 2:** Cutting force results in different machining conditions.

Exp	Machining Parameters	Tool State	Machining Environment	Cutting Force (N)	Temperature (°C)	Surface Roughness (µm)
1	V = 300 m/min, f = 0.05 mm/rev, doc = 0.1 mm	Fresh tool	MQL	50.53	234.7	0.387
2	V = 300 m/min, f = 0.05 mm/rev, doc = 0.1 mm	Fresh tool	Cryogenic (LN_2_)	91.23	149.8	0.244
3	V = 300 m/min, f = 0.05 mm/rev, doc = 0.1 mm	Worn tool	MQL	233.89	730.9	1.114
4	V = 300 m/min, f = 0.05 mm/rev, doc = 0.1 mm	Worn tool	Cryogenic (LN_2_)	170.6	631.8	0.586

## Data Availability

The original contributions presented in this study are included in the article. Further inquiries can be directed to the corresponding author.
